# Diversity of *Vibrio* spp in Karstic Coastal Marshes in the Yucatan Peninsula

**DOI:** 10.1371/journal.pone.0134953

**Published:** 2015-08-07

**Authors:** Icela Ortiz-Carrillo, Neyi Eloísa Estrella-Gómez, Marcela Zamudio-Maya, Rafael Rojas-Herrera

**Affiliations:** Facultad de Ingeniería Química, Universidad Autónoma de Yucatán, Periférico Norte km 33.5, Tablaje catastral 13615, Colonia Chuburná de Hidalgo Inn, C.P., 97203, Mérida, Yucatán, México; Beijing Institute of Microbiology and Epidemiology, CHINA

## Abstract

Coastal bodies of water formed by the combination of seawater, underground rivers and rainwater comprise the systems with the greatest solar energy flow and biomass production on the planet. These characteristics make them reservoirs for a large number species, mainly microorganisms. Bacteria of the genus *Vibrio* are natural inhabitants of these environments and their presence is determined by variations in the nutrient, temperature and salinity cycles generated by the seasonal hydrologic behavior of these lagoon systems. This study determined the diversity of the genus *Vibrio* in 4 coastal bodies of water on the Yucatan Peninsula (Celestun Lagoon, Chelem Lagoon, Rosada Lagoon and Sabancuy Estuary). Using the molecular technique of 454 pyrosequencing, DNA extracted from water samples was analyzed and 32,807 reads were obtained belonging to over 20 culturable species of the genus *Vibrio* and related genera. OTU (operational taxonomic unit) richness and Chao2 and Shannon Weaver diversity indices were obtained with the database from this technique. Physicochemical and environmental parameters were determined and correlated with *Vibrio* diversity measured in OTUs.

## Introduction

The genus *Vibrio* is the most diverse and abundant group of marine bacteria with 74 described species, and its taxonomy is under constant review due to the incorporation of genotypic and molecular analyses that show this genus to be highly heterogeneous [1,2]. The species of clinical importance are *V*. *cholerae*, *V*. *parahaemolyticus*, *V*. *vulnificus*, *V*. *alginolyticus*, *V*. *fluvialis*, *V*. *mimicus*, *V*. *hollisae*, *V*. *damsela*, *V*. *furnissii*, *V*. *cincinnatiensis*, *V*. *harveyi* and *V*. *metschnikovii*. There are also species of ecological and probiotic importance, such as *V*. *fischeri*, *V*. *splendidus*, *V*. *halioticoli*, *V*. *mediterranei* and *V*. *rotiferanius* [2,3].

Saline environments comprise the natural habitat of vibrios [4] and although their salt requirements fluctuate, the majority develop well between 2 and 2.5% salt. They can be found in the water column, sediments, zooplankton, plankton, algae and the gastrointestinal tract of many marine organisms [5–8]. Another special characteristic of this genus is the ability of its species to live under conditions of low salt and nutrient concentrations, by reducing their size to 0.2 μm (microvibrios) [7,9].

The members of this genus are highly specialized because they have the capacity to perform specific metabolic activities with respect to the use of carbon depending on the depth at which they are found. For example, *Vibrio profundum* presents optimal growth at 2,000 atmospheres and produces a large quantity of fatty acids that help it to maintain flow through the cell membrane at high pressures and low temperatures [9,10]. These bacteria are capable of degrading labile polymers such as starch, casein and agar [11] and they also produce a wide variety of enzymes, of which quinolase and chitinase are of particular significance because they provide the ability to degrade chitin and use it as a source of carbon and nitrogen [12–14].

Environmental gradients (temperature, salinity and nutrients) and biological factors influence the distribution and dynamics of *Vibrio* populations [5,12,15–17]. The species of genus *Vibrio* possess the versatility to develop in different environments and temperatures, however the optimum development for most species has been reported above 17°C. Some species are capable of “hibernating” in sediments or associating with marine fauna, which allows them to store a large quantity of proteins and develop biofilms, ensuring that they respond efficiently to the constant changes in ecosystems [2,18,19].

Coastal bodies of water are systems that are subjected to significant variability in their determining ecological factors due to a wide range of influences, including hydrologic, climatic and anthropogenic factors. On the Yucatan Peninsula these bodies of water are karstic in nature, which makes them rich in carbonates and other salts. Furthermore, water residency is not constant and therefore results in significant changes in salinity levels[20]. These special characteristics of the region influence the development, prevalence and distribution of the different organisms that inhabit these lagoons.

In these lagoons riverine tourism and fishing activities are widely practiced thus making an important issue to know which epidemiological and ecological important microbial species inhabit these systems. For example, octopus, which has become a highly important exportation product for Mexico, are caught in riverine areas under the influence of coastal lagoons, while other shellfish species like shrimp and oysters are directly extracted from lagoons being both octopus and shellfish intended for human consumption frequently raw or undercooked. As stated above, several *Vibrio* species are clinically, ecologically as well as biotechnologically important and, as far as we are aware, there are no reports on the taxonomic structure of these microorganisms that dwell in the coastal lagoons of the Yucatan Peninsula. Therefore, the aim of this work was to study the taxonomic structure and diversity of the genus *Vibrio* in 4 coastal lagoons by enrichment in selective medium strategy and subsequent massive 16S rRNA gene sequencing.

## Materials and Methods

### Study sites

Samples of surface water were taken from 4 coastal lagoons on the Yucatan Peninsula in Mexico: Celestun (20° 45' N—90°22' W), Chelem (21°15' N—89°45' W), Rosada Lagoon (21°19' N—89°19' W), and Sabancuy Estuary (18°58' N—91°12' W). The sampling was performed from August to October of 2011 (Chelem 08/24; Laguna Rosada 09/06; Celestún 09/28; Sabancuy 10/25). The sampling was random without replacement and 10 samples of surface water were collected along a transect parallel to the coastal axis. Samples were deposited in sterile plastic bottles and conserved in refrigeration at 4°C. All samples were processed within 24 hours after sampling.

According to the Mexican laws and regulations no permissions are required to obtain water and sediment samples from open public areas.

### Analysis of environmental and physicochemical parameters

Determinations of the environmental parameters were performed with a Hach 5465000 model 156 multi-parameter measuring instrument. The Lorenzen method was used to determine chlorophyll-a [21] with 90% acetone and the concentration was calculated according to the formula:

$$Chla=\;27.63\frac{\left(\text{OD}665\text{o }-\text{ OD}665\text{a}\right)\left(\text{VA}\right)}{\text{VM}}\text{x L}$$

Where, OD665o: absorbance at 665 nm before acidification; OD665a: absorbance at 665 nm after acidification; VA: volume (ml) of acetone for extraction; VM: volume (ml) of filtered water; L: length (cm) of the photometric cell.

Determinations of the physicochemical parameters (silicates, phosphates, nitrates, nitrites and ammonia) were performed using the spectrophotometric techniques described and modified by Strickland and Parsons [21].

### Enrichment

The water samples were cultured in trypticase soy broth (TCS) (BD-Bioxon) containing 0.5% NaCl. Additionally, the samples enriched in salt medium were cultured in TCS supplemented with 3% NaCl. All cultures were incubated at 37°C with agitation at 150 rpm for 48 hours.

### Total (metagenomic) DNA extraction

0.5 ml of overnight culture was poured into a 1.7 ml microcentrifuge tube with the addition of 1 ml of TEN buffer (100 mM Tris– HCI, 50 mM EDTA, 500 mM NaCl, pH 8.0) and vortexed for 1 min. After centrifugation for 10 min at 10,000 xg at RT, the pellet was resuspended in 1 ml of TEN buffer with 0.2 mg of added lysozyme and incubated for 1 h at 37°C under agitation. Then three cycles of freezing/defrosting were done by incubation 10 min in an ice/alcohol bath and then 5 min at 65°C. 100 μl of 20% SDS was added and vortexed for 1 min and incubated for 30 min at RT. After centrifugation for 10 min at 10,000 xg at RT, the supernatant was transferred into a fresh microcentrifuge tube and 500 μl of 5 M potassium acetate was added and then incubated at 65°C for 5 min. Tubes were placed in an ice bath for 20 min and centrifuged at 20,000 xg for 30 min at 4°C. The supernatant was transferred into a fresh microcentrifuge tube and 200 μl of a 4% silica suspension was added and incubated at RT for 2 min with gentle agitation. Silica-DNA complex was recovered by centrifugation at 16,000 xg for 2 min at RT and the pellet was washed by adding 1 ml of 70% ethanol. DNA was eluted with 50 μl of sterile distilled water and incubated at 55°C for 5 min and recovered by centrifugation at 16,000 x g for 5 min at RT [22]. DNA samples were quantified with a NanoDropND-1000 spectrophotometer (NanoDrop Technologies,Wilmington, DE, USA).

### Sample pooling

Since a great number of subsamples were generated per lagoon (20 from each) we decide to prescreen samples to reduce the total number of sequencing reactions. For that we used a DGGE approach based on the technique described by Muyzer et al. [23], using specific primers for the genus *Vibrio*, GC567f and 680r [24], to visually analyze if meaningful differences existed among different samples from the same lagoon. After DGGE experiments it was shown there were not major differences among enriched subsamples of the same lagoon (data not shown), so we decided to pool together five DNA samples of each enrichment condition per lagoon what resulted in two composite samples for every lagoon. Data presented in this paper correspond to those composite samples.

### Massively parallel bTEFAP

Purified metagenomic DNAs were submitted to the Research and Testing Laboratory (RTL) (Lubbock, TX, USA) for 16S rRNA tag-pyrosequencing. Bacterial tag-encoded FLX amplicon pyrosequencing (bTEFAP) was performed as described previously using Gray28F (5’-TTTGATCNTGGCTCAG-3’) and Gray519r (5’-GTNTTACNGCGGCKGCTG-3’) were used for amplification of the variable regions V1-V3 [25]. Initial generation of the sequencing library utilized a one-step PCR with a total of 30 cycles, a mixture of HotStart and HotStar high fidelity Taq polymerases, and amplicons originating and extending from the 28F for bacterial diversity. Tag-encoded FLX amplicon pyrosequencing analyses utilized Roche 454 FLX instrument with Titanium reagents; Titanium procedures were based on RTL protocols (www.researchandtesting.com).

### Data analysis, bacteria identification and diversity index

Following sequencing, all failed sequence reads, low quality sequence ends and tags and primers were removed and sequences collections depleted of any non-bacterial ribosome sequences and chimeras using B2C2 [26], as has been described previously [25,27]. To determine the identity of bacteria in the remaining reads, sequences were denoised, assembled into clusters and queried using a distributed BLASTn. NET algorithm [28] against a database of high quality 16S bacterial sequences derived from NCBI. Database sequences were characterized as high quality based upon similar criteria utilized by RDP. Using a.NET and C# analysis pipeline. The resulting BLASTn outputs were compiled, validated using taxonomic distance methods, and data reduction analysis performed as described previously [25,27]. Based upon the above BLASTn derived sequence identity (percent of total query sequence length which aligns with a given database sequence) and validated using taxonomic distance methods, the sequences were classified at the appropriate taxonomic levels based upon the following criteria: sequences with identity scores (relative to known or well characterized 16S sequences) greater than 97% identity (<3% divergence) were resolved at the species level, between 95% and 97% at the genus level, between 90% and 95% at the family and between 85% and 90% at the order level, 80 and 85% at the class and 77% to 80% at the phylum level. In addition, the high-score pair must be at least 75% of the query sequence or it will be discarded, regardless of identity.

Sequencing reads were aligned and clustered following the Ribosomal Database Project (RDP-Release 10) pyrosequencing pipeline (http://pyro.cme.msu.edu/). Shannon, Chao2, and evenness indices, as well as rarefaction curves, Jaccard index and heat map were obtained using the RDP tools.

### Accession number

All 16S rRNA gene sequences were deposited in the SRA database at the National Center for Biotechnological Information (http://www.ncbi.nlm.nih.gov/sra) with accession number SRA149316 (Experiments SRX502094 (CECS), SRX502095 (CESS), SRX502096 (CHCS), SRX502097 (CHSS), SRX502098 (LRCS), SRX502099 (LRSS), SRX502100 (SACS) and SRX502101 (SASS)).

### Correlation analysis

The Pearson coefficient was used to correlate the environmental and physicochemical parameters with diversity measured in operational taxonomic units (OTUs). They were analyzed by means of multivariate correlation analysis with ρ ≤ 0.05 using the STATGRAPHIC CENTURION XVI package.

## Results

Pyrosequencing of the water samples cultured in trypticase soy broth enriched with NaCl yielded a total of 46,827 reads from which 32,807 reads (70.1%) passed the quality control stage. These reads were grouped according to lagoon and culture conditions. The genus *Vibrio* was present in 43.7% (14,337 reads), the related genera such as *Aliivibrio*, *Photobacterium* and *Salinivibrio* in 7.4% (2,420 reads) and other genera in 48.9% (16,050 reads).

This database permitted the calculation of the alpha diversity of each site, using the number of OTU's (sequence similarity cut-off 0.97) and the Shannon-Weaver and Chao2 diversity indices. Based on these values, the most diverse sites were determined to be Rosada Lagoon and Celestun Lagoon. Celestun Lagoon yielded richness values of 116 OTU's, a Shannon index of 3.0 and a Chao2 index of 126.5. Rosada Lagoon showed a richness of 132 OTU’s, a Shannon index of 3.7 and a Chao2 index of 143.1 (Table 1).

**Table 1:** Number of reads and diversity indexs of *Vibrio* spp for each composite sample.

	No. of reads						
Samples	*Vibrio*	Related genera*	Other genera	Total	OTU’s**	H’	chao2
**CECS**	1025	558	3660	5243	107 (30)	2.3	115.5
**CESS**	1582	94	1591	3267	116 (51)	3.0	126.5
**CHCS**	3447	455	1063	4965	107 (78)	3.1	115.7
**CHSS**	2035	329	2047	4411	90 (44)	3.1	102
**LRCS**	1631	206	1368	3205	112 (57)	3.6	113.6
**LRSS**	1973	318	1414	3705	132 (62)	3.7	143.1
**SACS**	2144	362	1347	3853	107 (65)	3.4	112.2
**SASS**	500	98	3560	4158	98 (14)	2.7	102.5
**TOTAL**	14337	2420	16050	32807	-	-	-

*Refers to *Aliivibrio*, *Photobacterium* and *Salinivibrio* genera

**Sequence similarity cut of 0.97. Numbers in parenthesis represent *Vibrio* OTU´s

The analysis of sequences from the lagoons with most diversity index without enrichment showed that the community of Celestun was dominated by *Geobacillus* and *Pseudomonas*, representing over 70% of total reads whereas *Vibrio* represented only 0.04%. On the other hand, in Rosada Lagoon a more structured community was found and prevalent genera were *Marinobacterium* (26%), *Pseudoalteromonas* (15.5%), *Synechococcus* (7.8%), *Ruegeria* (7.2%), *Bacillus* (6.7%) and *Planktaluna* (6.6%) While *Vibrio* represented the 0.29% of total reads (data not shown).

**Fig 1:**
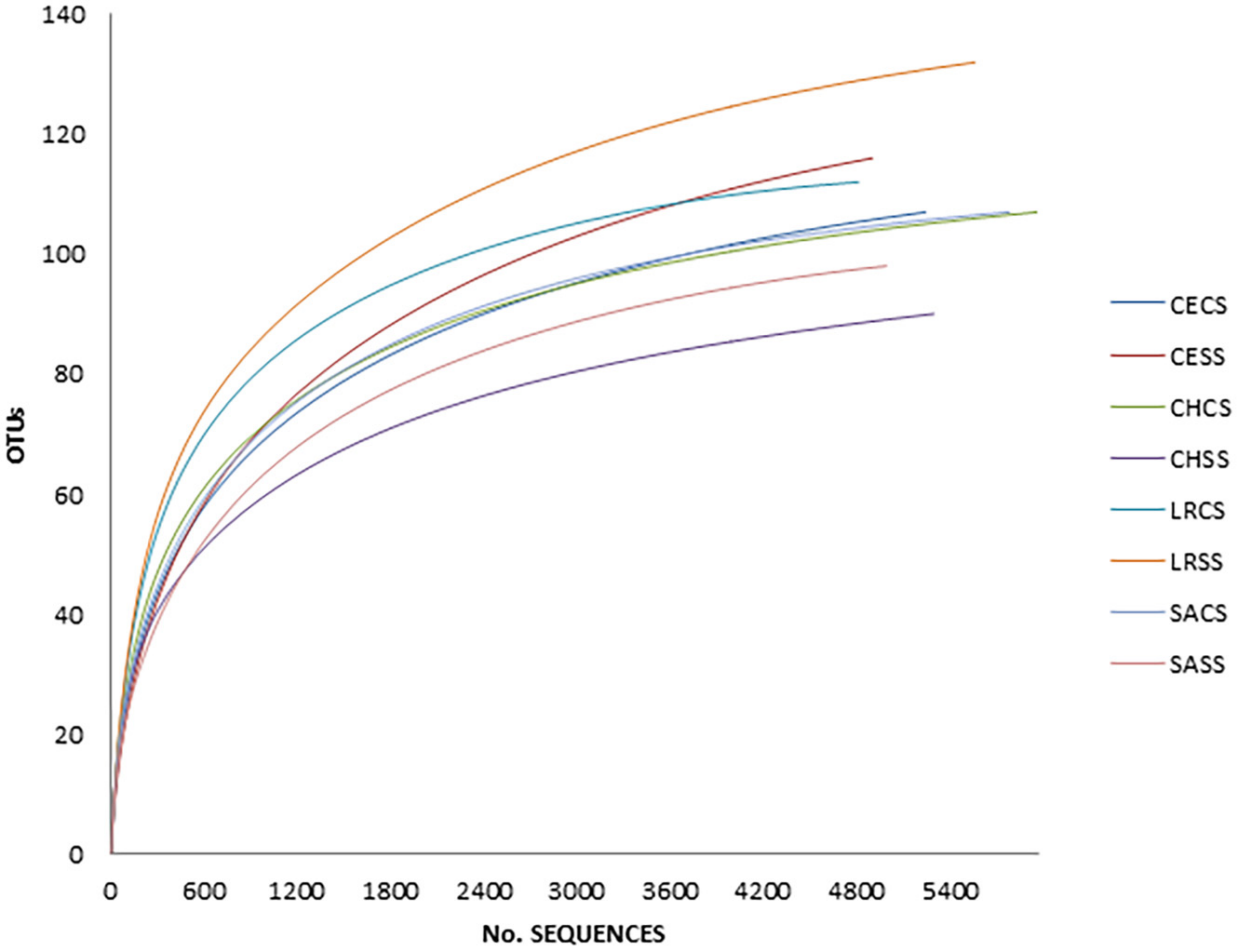
Rarefaction curve at 0.97 similarity of water samples from 4 coastal lagoons: Celestun (CE), Chelem (CH), Laguna Rosada (LR) and Sabancuy (SA); in the two culture conditions: CS (water samples grown tryptone soy broth + 3% NaCl) and SS (water samples cultured in tryptone soy broth).

Rarefaction analysis of the study sites (enriched samples) established that the sampling conditions were representative for determining the total diversity of the genus *Vibrio*. For each site studied, a rarefaction curve reached an asymptote at over 4,000 reads (Fig 1).

**Fig 2:**
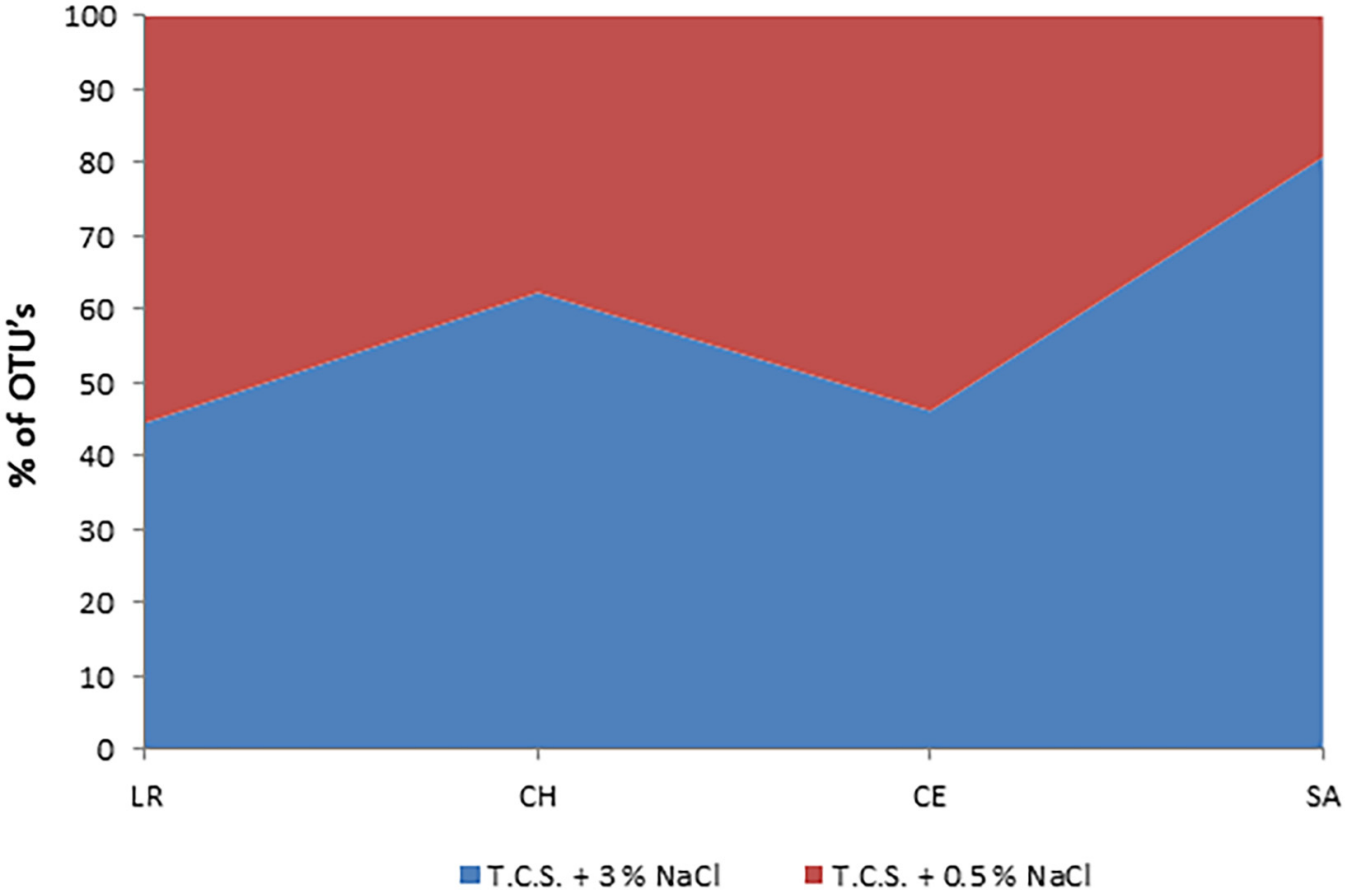
Halophilicity of *Vibrio* species in the four coastal lagoons analyzed: Rosada lagoon (LR); Chelem (CH); Celestun (CE) and Sabancuy (SA). + NaCl (T.C.S. broth + 3% NaCl);—NaCl (T.C.S.). % of OUT’s is referred to the total number of OTU´s classified within the genus *Vibrio* for each lagoon

It was possible to confirm the halophilicity of the genus *Vibrio* by enrichment under two different NaCl concentrations. This characteristic allowed for the evaluation of the development of the genus *Vibrio*, with preferences for sodium, at sites with different salinity conditions (Fig 2).

**Fig 3:**
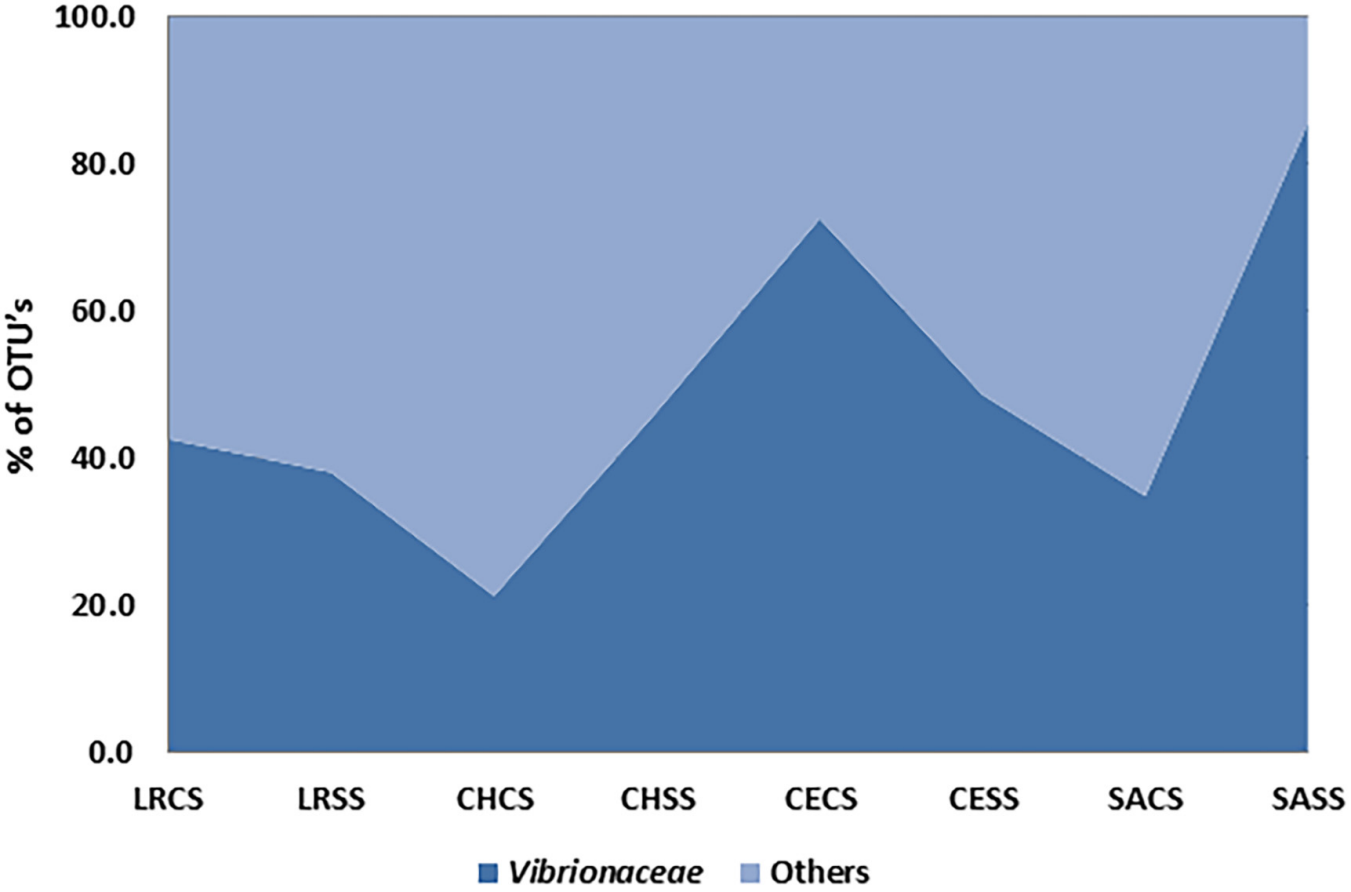
Effectiveness of the culture medium for the isolation of the family *Vibrionaceae* in four coastal lagoons: Rosada lagoon (LR); Chelem (CH); Celestun (CE) and Sabancuy (SA). CS (water samples cultured in tryptone soya broth + 3% NaCl). SS (water samples cultured in tryptone soy broth).

Over 50% of the OTU’s were found to belong to the *Vibrionaceae* family, which confirms the effectiveness of culturing *Vibrio* in TCS broth suplemented with NaCl. This effectiveness was clearly observed at sites where salinity was low, such as Celestun Lagoon, where a value of over 60% of OTU’s was obtained (Fig 3). Genera like *Fusobacterium* (1–20%), *Cetobacterium* (5–35%) and *Clostridium* and *Shewanella* (1–10%) were also identified in the enriched samples.

**Fig 4:**
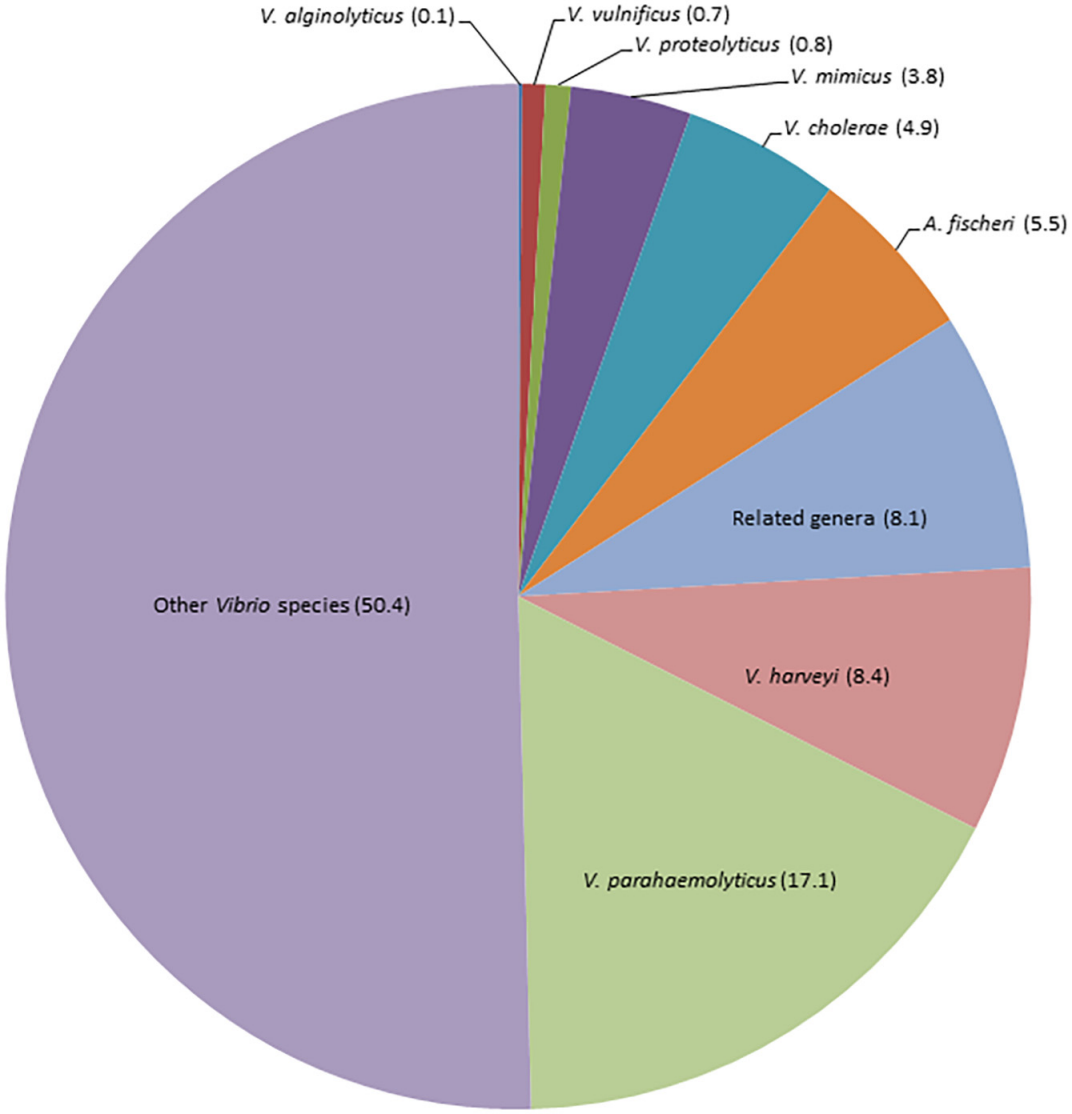
Prevalence of *Vibrio* spp in coastal lagoons of the Yucatan Peninsula.

At the species level, pathogenic species such as *V*. *cholerae* (4.9%), *V*. *parahaemolyticus* (17.1%), and *V*. *vulnificus* (0.7%) were identified; as well as others of ecological importance such as *V*. *mimicus* (3.8%), *V*. *harveyi* (8.4%), *V*. *proteolyticus* (0.8%), *V*. *fischeri* (5.5%) and *V*. *algynolyticus* (0.1%). Unclassified *Vibrio* species represented 50.4% (Fig 4).

**Fig 5:**
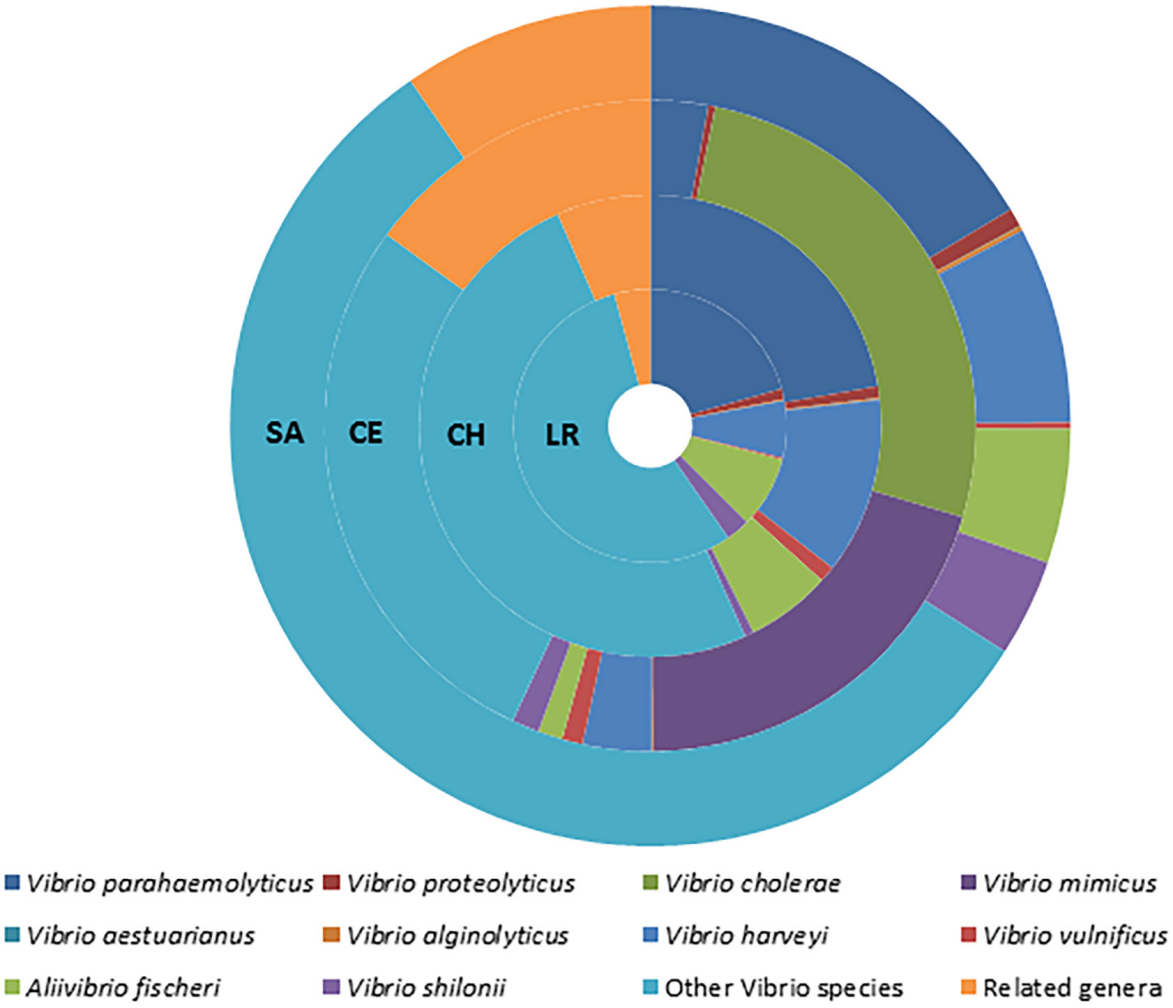
Taxonomic structure of the genus *Vibrio* in the four coastal lagoons studied: Rosada lagoon (LR) Chelem (CH); Celestun (CE) and Sabancuy (SA).

Different *Vibrio* species were identified at all sites. Very particular species were identified for each site, such as *V*. *cholerae* and *V*. *mimicus* in Celestun; *V*. *harveyi* in Chelem, and *V*. *fischeri* in Rosada Lagoon. However, the prevalence of some species was also observed in more than one lagoon, such as *V*. *parahaemolyticus* and *V*. *vulnificus* present in Rosada Lagoon and Chelem (Fig 5).

**Fig 6:**
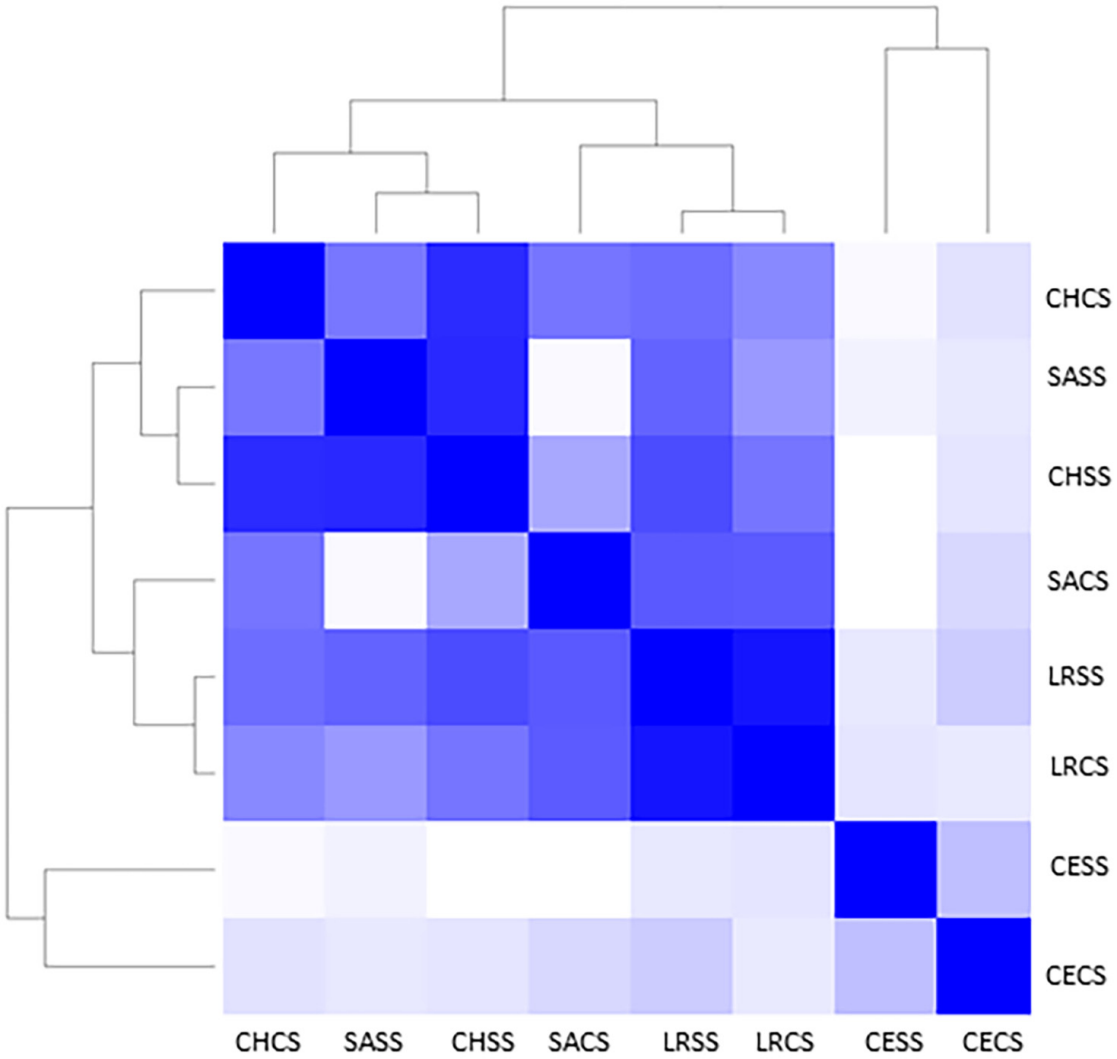
Heat map based on Jaccard at 0.97 similarity of the prevalence of *Vibrio* spp in the 4 coastal lagoons: Chelem (CH), Sabancuy (SA), Laguna Rosada (LR) and Celestun (CE) in two culture conditions: tryptone soy + 3% NaCl (CS) and tryptone soya (SS).

In the study of beta diversity, the taxonomic dissimilarity between the different lagoons was determined, and sites that are geographically distant from each other or samples enriched under different conditions were found to present similarities between their *Vibrio* communities. This was the case with Sabancuy and Chelem lagoons, where a similar structure of *Vibrio* community was observed, despite the fact that they are very distant in geographical terms. Also in one site (Rosada Lagoon), the species found were observed to be highly similar for both samples enriched under different conditions. This similarity analysis can be seen in the heat map in Fig 6, where the color intensity shows the similarity between the species present.

Correlations between the diversity of the genus *Vibrio* and environmental and physicochemical parameters were performed by multivariate analysis. The Pearson coefficient was obtained, establishing ρ ≤ 0.05 as a significant positive value. Celestun showed a significant positive correlation between the number of OTU’s and salinity (ρ = 0.0449). Likewise, Rosada Lagoon presented a significant positive correlation between the number of OTU’s and chlorophylls (ρ = 0.0255) (S1 Table). (http://doi.pangaea.de/10.1594/PANGAEA.847579).

## Discussion

The Yucatan Peninsula is characterized by having estuary-lagoon systems that are shallow, with highly variable water residence times and salinity, generating high species diversity, especially of microorganisms [20]. This work comprises the first study of the composition of the taxonomic structure of the *Vibrio* community in the coastal lagoons of the Yucatan Peninsula. This genus is considered to be of great epidemiological and ecological importance.

Species diversity was determined by massive sequencing of metagenomics DNA obtained from enriched water samples. This tool produced a database that allowed to identifying the species that inhabit each of the lagoons and describing the taxonomic structure of the *Vibrio* community dwelling in these bodies of water. This information revealed that Rosada Lagoon was the most diverse site, yielding the highest values for the number of OTU’s and the Shannon and Chao2 diversity indices under the two culture conditions. This lagoon is a system characterized by having the highest salinity levels and being the least polluted of the studied sites and receives little influence from humans [20]. The high diversity of OTU’s can be caused by the fact that it is a hypersaline system, therefore providing many *Vibrio* species with the appropriate conditions for development. A large number of species from this genus need Na^+^ to grow and develop, and usually display optimum growth rates between 0.5 and 3.5% NaCl [2,8,16,29]. An example of this is *V*. *parahaemolyticus*. Its presence in this lagoon is because this system meets the salinity conditions required for its development, which are above 3.0% salt (S1 Table). This species is of great epidemiological importance, given that it causes severe gastroenteritis in humans if raw or undercooked contaminated seafood is consumed [4].

Species of great ecological importance were also identified in Rosada Lagoon, such as *V*. *fischeri*, which is a species that presents an interesting symbiosis with the squid *Euprymna scolopes*, providing it with bioluminescence thanks to the expression of the *lux* operon [29]. Another species of ecological as well as pathogenic importance is *V*. *harveyi*, a bioluminescent bacterium that produces a large quantity of degradative enzymes. However, pathogenic strains of this species have caused severe economic losses in aquaculture through the contamination of oysters and shrimp [19].

In Rosada Lagoon, a positive correlation was observed between OTU’s and chlorophyll-a. These data agree with other studies that show positive correlations between the diversity of the genus *Vibrio* and chlorophylls, in which the genus *Vibrio* has been observed to attach to phytoplankton, algae and zooplankton present in bodies of water, establishing a symbiosis between these organisms [3,5,8,30].

Not all of the species are halophiles, however, and some of them can develop under very low salinity conditions. These conditions are met by Celestun Lagoon, which is a mesohaline system caused by the effect of its geographical location on the “ring of cenotes” on the salinity gradients in its different zones [31]. This lagoon also yielded high values for species richness and diversity indices. *V*. *cholerae* was identified, which is the species that is the etiological agent of cholera and has serotypes and biotypes that have caused 7 epidemics around the world [32]. The highest percentages of this species from among the four study sites were found in this lagoon. The same applied to *V*. *mimicus*, which is the species that shares the greatest number of genotypic and phenotypic characteristics with *V*. *cholerae* and can cause severe gastroenteritis [33]. The hemolysins that it produces are very similar to those from pathogenic strains such as *V*. *cholerae* El Tor and the thermostable direct hemolysin (TDH) of *V*. *parahaemolyticus*. For this reason it is thought that *V*. *mimicus* could become an emerging pathogen [34]. *V*. *cholerae* and *V*. *mimicus* were identified only in this lagoon, given that their salinity requirements are minimal and they can even develop in fresh water. Celestun is also a lagoon subject to a strong anthropogenic influence as a result of touristic activities, causing it to become polluted [20]. Other species of epidemiological importance identified in this lagoon, were *V*. *parahaemolyticus*, *V*. *fluvialis*, *V*. *harveyi* and *V*. *porteresiae* in samples enriched in TCS + 3% NaCl. OTU’s identified in this sample were positively correlated to salinity, which agree with other studies that show positive correlations between salinity and the diversity of the genus *Vibrio* [15,16,35,36]. Other species, such as *V*. *natriegens*, which is capable of fixing atmospheric nitrogen into more available forms such as ammonia [37] were also identified. *V*. *fluvialis* was detected at a very low percentages in this lagoon; this bacterium has been reported to cause extraintestinal infections such as peritonitis, hemorrhagic cellulitis, bacteremias, cerebritis and otitis [38]. Other species found were V. *vulnificus*, present in oysters and reported as a human pathogen [39]; and *V*. *shilonii*, synonymous with *V*. *mediterranei*, and recognized as the causal agent of bleaching of the coral *Oculina patagonica* [40].

The genus *Vibrio* is a group of very cosmopolitan bacteria that develop in both freshwater systems (rivers and lakes) and in saline systems (seas or coastal bodies of water) [8]. At our studied sites, the levels of the environmental and physicochemical parameters are conditions that can vary depending on the season and geographical location, and these conditions influence the development of the different species of this genus.

Chelem Lagoon is considered the most polluted lagoon as a result of anthropogenic influences and is currently undergoing an eutrophication process [20,31]. It is surrounded by human settlements, and is also subject to significant touristic and fishing activity. In this lagoon, it was observed that the addition of 3% NaCl favored the most halophilic species, such as *V*. *parahaemolyticus*, for which it yielded the highest value of all of the studied sites. As mentioned previously, this species causes severe gastroenteritis in humans, and a large number of infections caused by this species have been reported on the coast of the Gulf of Mexico [41]. *V*. *vulnificus* another species of pathogenic importance for humans was also identified in this lagoon. This bacteria has a very fast-acting pathogenic mechanism, and in the United States 303 cases of infections were reported from 2000 to 2009, 148 of which were fatal [39]. Although low percentages of this species were reported at Chelem, its presence should not be ignored given the riverine fishing activity that takes place in this site. The same applies to *V*. *parahaemolyticus*. *V*. *harveyi* was detected in high number under both enrichment conditions. This species is considered a pathogen of some species of mollusks and fishes, especially shrimp [2,42]. This lagoon showed the highest values among all of the lagoons for OTU’s from this species, as well as for *V*. *natriegens* and *V*. *fischeri*, Finally, the highest assignation of reads to unclassified *Vibrio* (*Vibrio* spp) were for samples obtained from Chelem and enriched in TCS with 3% NaCl.

The fourth studied site, Sabancuy Estuary, is the most geographically distant from the other three sites and is located in the state of Campeche. It is considered an estuary, fed by the Terminos Lagoon and the sea [43]. It has variable salt concentrations. It yielded high OTU values for samples enriched in TCS with 3% NaCl which might indicate that these salinity variations may favored the development of species that are salt-dependent. In this lagoon, species such as *V*. *parahaemolyticus*, *V*. *vulnificus*, *V*. *fischeri* and *V*. *shilonii* were detected and a very high percentage of *Vibrio* spp obtained. *V*. *cholerae* and *V*. *mimicus* were not found in these samples explainable by reasons mentioned above. Other species such as *V*. *neptunius*, *V*. *proteolyticus*, *V*. *sinaloensis* were also observed in very small percentages.

### Rarefaction and cluster analysis

Three main approximations for quantifying diversity were used to analyze *Vibrio* communities: species richness estimators, diversity indices and rarefaction analysis [44]. The latter was used to evaluate the “sampling effort” to quantify the diversity of the genus *Vibrio* at each site. This analysis related the number of base sequences with the number of OTU’s obtained, and from this relationship, a curve is obtained indicating that the total diversity has been sampled when it reaches an asymptote. These asymptotes were found to be greater than 4,000 for all of the sampled sites which means that sampling was representative in order to quantify the diversity of this genus at all the sites.

Cluster analysis showed the similarities and differences between the different *Vibrio* communities, based on the site and culture condition. This analysis was performed with the Jaccard index set at 0.97 similarity. The communities of vibrios of Sabancuy and Chelem (samples enriched in TCS with no added salt), despite the fact that the two sites are very different and geographically distant, are quite similar perhaps because these two lagoons have very similar salinity and other ecological parameters caused by the anthropogenic influence (both lagoons are surrounded by human settlements).

In the same way, *Vibrio* community of Rosada Lagoon showed very little differences in samples enriched with or without salt. It would seem that the addition of NaCl does not make a difference in terms of species proliferation maybe due to the fact that it is a hypersaline lagoon and autochthonous species are halophilic, such as *V*. *parahaemolyticus* and *V*. *harveyi*.

Celestun showed significant differences with respect to the other sites and grouped separately. Many of the species found at this site are very particular. For example, we found *V*. *cholerae* in very high percentages and *V*. *mimicus* in a lower abundance; none of these species were found in any other sample from other lagoon.

## Conclusions

Knowledge of epidemiologically relevant bacterial groups such as *Vibrio*, in coastal environments where activities of fishing and tourism take place, is of crucial importance. As it was demonstrated in this study, the taxonomical structure of *Vibrio* communities in lagoons of the Yucatan Peninsula seems to be quite complex and variable and no significant correlation was found with environmental parameters except salinity and chlorophyll in particular lagoons. Besides, *Vibrio* species that may pose a threat to people who eat fish and shellfish extracted from these water bodies were detected. Moreover, the presence of species of biotechnological interest, as well as other species that might play a role in the ecosystem was evidenced. In this respect, the presence of species such as *V*. *harveyi*, which produce a large amount of degradative enzymes, may be indicative of an increase in the levels of contamination of these bodies of water, or because of their status of marine heterotrophic bacteria they could be acting as regulators of the major biogeochemical cycles that develop in these environments. These studies are particularly important for regions like the Yucatan Peninsula, given the vastness of coastal lagoons (ca. 19000 km^2^) and the influence they may have on extensive marine areas of the Gulf of Mexico.

## Supporting Information

S1 Table Physicochemical parameters of water sample the four coastal lagoons. (DOCX)
