# Supplementary material for: Diversity of Vibrio spp in Karstic Coastal Marshes in the Yucatan Peninsula
Source: PLoS One. 2015 Aug 7;10(8):e0134953. doi: 10.1371/journal.pone.0134953 (PMC4529161; doi:10.1371/journal.pone.0134953)
Supplement: S1 Table — (DOCX) [file pone.0134953.s001.docx]

| LAGOON (SAMPLE) | LAGOON  TYPE | SITE LOCALIZATION | SiO_4_  (µM) | PO_4_ (µM) | NH_4_  (µM) | NO_3_  (µM) | NO_2_ (µM) | Chl. A  (µg.l^-1^) | pH | SAL. (%) | TEMP.  (ᵒC) |  |
| --- | --- | --- | --- | --- | --- | --- | --- | --- | --- | --- | --- | --- |
| Celestún | oligohaline | 20°45’N- 90°22’W | 4164.5±449.76 | 1.49±0.38 | 16.69±12.05 | 10.29±3.76 | 0.50±0.19 | 48.42±33.43 | 7.99±0.22 | 0.87±0.40 | 30 |  |
| Chelem | hyperhaline | 21°15’N- 89°45’W | 698.3±674.27 | 1.43±0.59 | 15.85±24.40 | 1.77±1.18 | 0.32±0.38 | 40..61±25.02 | 8.24±0.12 | 3.06±0.61 | 31 |  |
| Rosada | hyperhaline | 21º19’N- 89º19’W | 1286.3±583.54 | 0.67±0.70 | 11.12±3.98 | 2.24±0.86 | 0.09±0.09 | 18.25±9.10 | 8.03±0.05 | 3.46±0.21 | 28 |  |
| Sabancuy | estuarine | 18°58´N- 91°12’W | 453.2±431.04 | 1.71±1.15 | 22.06±6.92 | 2.77±1.88 | 0.03±0.03 | 79.01±13.71 | 8.06±0.24 | 2.65±0.33 | 32 |  |
